# Isolated pulmonary valve infective endocarditis in a middle aged man caused by *Candida albicans:* a case report

**DOI:** 10.1186/s12879-014-0557-5

**Published:** 2014-10-30

**Authors:** Sudhakar Devathi, Bryan Curry, Saumil Doshi

**Affiliations:** Division of Infectious Diseases, Howard University College of Medicine, Washington, DC USA; Division of Cardiology, Howard University College of Medicine, Washington, DC USA

**Keywords:** Pulmonary valve, Candida albicans, Fungal endocarditis

## Abstract

**Background:**

Pulmonary valve endocarditis without the involvement of other valves represents 1.5-2% of all cases of infective endocarditis. Isolated pulmonary valve endocarditis caused by *Candida* is extremely rare with only one reported case in the literature and none reported in the United States. Guidelines for management of *Candida* endocarditis recommend a combination of medical and surgical therapy.

**Case presentation:**

A 61-year-old homeless male presented with fever, cough and shortness of breath. He was urgently intubated for hypoxia. He was initially diagnosed with pneumonia but did not improve with empiric antibacterial therapy. *Candida* species were isolated from bronchoalveolar lavage fluid and the patient eventually developed persistent *C. albicans* bloodstream infection. On further workup he was found to have infective endocarditis with a large vegetation across the pulmonary valve. No other valves were involved. He was treated with intravenous antifungal therapy for eight weeks. Valvular surgery was not performed. Follow up echocardiography after completion of therapy did not show any vegetations and the patient clinically improved.

**Conclusion:**

This is the second reported case of isolated pulmonary valve endocarditis caused by *Candida* and the first to be successfully managed with antifungal therapy alone. Pulmonary valve endocarditis should be considered in cases of pneumonia with *Candida* and persistent fungemia. While surgery should be considered in all cases of *Candida* endocarditis, cure may be achieved with antifungal therapy alone.

**Electronic supplementary material:**

The online version of this article (doi:10.1186/s12879-014-0557-5) contains supplementary material, which is available to authorized users.

## Background

Pulmonary valve endocarditis without the involvement of other valves represents 1.5-2% of all cases of infective endocarditis [1]. Fungal endocarditis is also rare, comprising <10% of all endocarditis cases [2]. Despite the development of newer antifungal agents, mortality from *Candida* endocarditis is estimated to be 30-37% [3]-[5]. The Infectious Diseases Society of America 2009 candidiasis guidelines recommend a combined medical and surgical approach for treatment of *Candida* endocarditis [6]. There has been only one reported case of isolated pulmonary valve endocarditis with *Candida* which was managed surgically [7]. We report a case of isolated pulmonary valve endocarditis due to *Candida* managed with medical therapy alone.

## Case presentation

**Figure 1:**
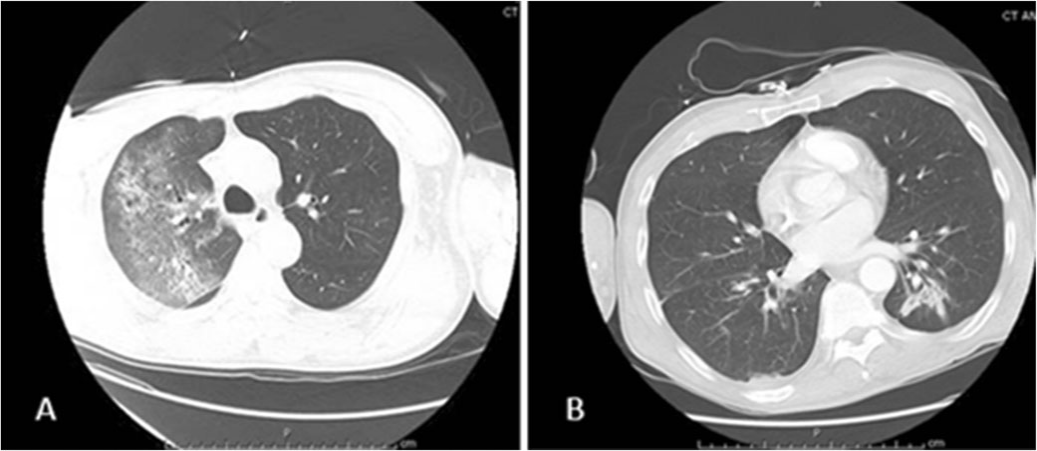
**Computed tomography of the chest. A**. Patchy opacities inthe right lung. **B**. Resolution of the opacities after intravenous Amphotericin B.

A 61 year-old homeless African-American male was admitted to the intensive care unit with fever, dyspnea, and hemoptysis for four days. He had a history of depression and active intravenous drug use. Physical examination was positive for crackles in both lung fields. He had no track marks on his skin. His white blood cell (WBC) count was 0.3 × 10^3^/μL with absolute neutrophil count 100/mm^3^. Urine toxicology screen was positive for opiates and benzodiazepines. His basic metabolic panel was normal but his arterial blood gas (ABG) analysis revealed a PaO_2_ of 62 mm Hg on room air. He was intubated in the emergency room for hypoxia. Computed tomography (CT) scan of the chest revealed large patchy opacities in the right upper and middle lobes consistent with pneumonia and no evidence of pulmonary embolism (Figure 1A). Empiric vancomycin and piperacillin-tazobactam were started. His initial blood cultures and serum HIV ELISA antibody were negative. Three tracheal aspirate specimen smears were negative for acid-fast bacilli.

**Figure 2:**
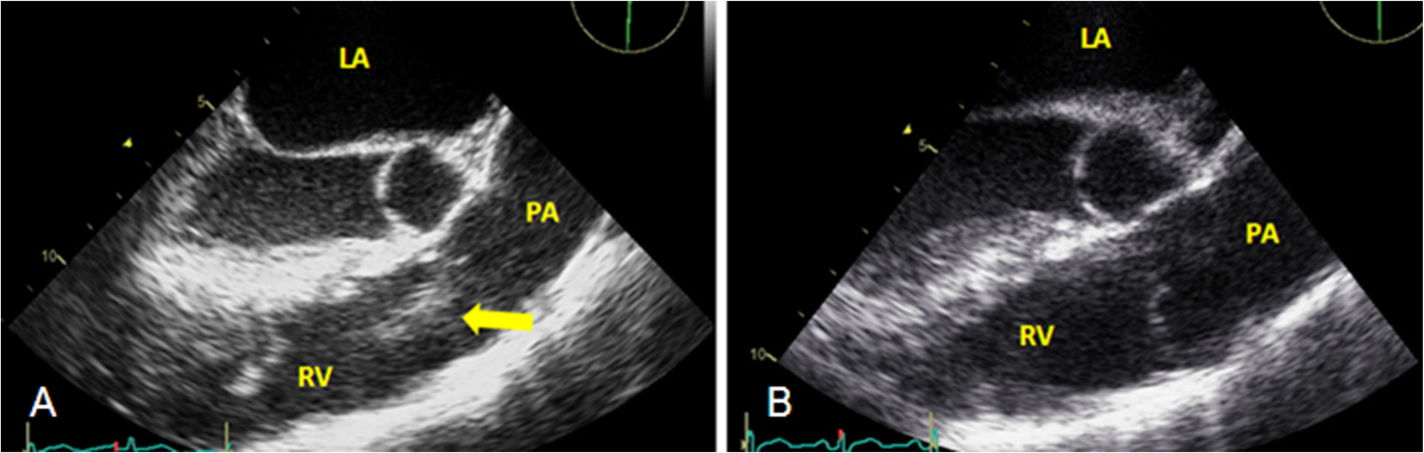
**Transesophageal echocardiogram. A**. Pulmonary valve vegetation **B**. No vegetation after four weeks of Amphotericin B.

Bronchoscopy performed on hospital day three showed black secretions from the right lower lobe. He was started on Micafungin after bronchoalveolar lavage cultures grew *Candida albicans* and *Candida glabrata*. Trans-bronchial biopsy was not performed due to friable mucosa. Trans-thoracic echocardiogram on hospital day five revealed no valvular abnormalities or vegetations. Because of persistent fever and hypoxia, a second bronchoscopy was performed on hospital day eight, which still showed black secretions; bronchoalveolar lavage cultures grew *Candida glabrata*. Two sets of blood cultures collected on hospital day twelve grew *Candida albicans.* A central venous catheter that had been placed on admission was removed; culture of the catheter tip showed no growth. Amphotericin B lipid complex was initially added to Micafungin. After his creatinine rose from 0.9 to 4.5 mg/dL, this was switched to Liposomal Amphotericin B; his creatinine normalized to 1.1 mg/dL by hospital day thirty. Additionally, his WBC count rose to 4.9 × 10^3^/μL with 68% neutrophils calculated on an automated differential with no interventions other than treating his underlying sepsis. The patient defervesced three days after amphotericin B was added and Micafungin was discontinued. However, repeat blood cultures on hospital day fourteen again grew *Candida albicans*. A trans-esophageal echocardiogram (TEE) performed on hospital day sixteen showed a 1.5 cm mobile mass on the pulmonary valve extending from the right ventricular outflow tract across the pulmonary valve into the pulmonary artery (Figure 2A).

A diagnosis of isolated pulmonary valve endocarditis secondary to *Candida* was made. Cardiothoracic surgery was consulted; they advised medical therapy and later re-evaluation as the patient was a high-risk candidate for surgery. Blood cultures obtained on hospital day 16 had no growth. After a tracheostomy was performed, the patient was transferred to a nursing home to complete liposomal amphotericin B for a total of eight weeks. His repeat CT chest at the time of discharge showed complete resolution of pulmonary infiltrates (Figure 1B) and he was weaned to tracheal mask. Lifelong suppressive oral fluconazole therapy was recommended. After eight weeks of antifungal therapy, a repeat TEE did not show any pulmonary valve vegetation (Figure 2B). The patient clinically improved, his tracheostomy was closed, and he was discharged back to the shelter. Six months after discharge, the patient was asymptomatic and doing well. He had stopped taking his oral fluconazole despite recommendations for lifelong suppressive therapy.

## Discussion

This report illustrates an unusual case of isolated pulmonary valve endocarditis with *Candida* in an intravenous drug user that was managed successfully with medical therapy alone. Isolated pulmonary valve endocarditis is rare [8]. Endocarditis in an intravenous drug user usually involves the tricuspid valve, but a structurally normal pulmonic valve is rarely the only one affected [9]. Possible reasons for this are twofold. First, the lower pressure gradient across the pulmonic valve leads to less shear stress compared to the other valves [10],[11]. This causes less valvular damage, making it less vulnerable for endocarditis. Second, valvular abnormalities (congenital or acquired) are less common in the pulmonary valve.

An autopsy analysis of nine cases of isolated pulmonary valve infective endocarditis revealed seven cases had congenital heart disease and five cases had septic pulmonary emboli [12]. It is unclear if our patient had a primary *Candida* pneumonia leading to endocarditis or a *Candida* blood stream infection with endocarditis and septic pulmonary emboli. We do not have a trans-bronchial biopsy to confirm a diagnosis of *Candida* pneumonia, which is by itself, is rare [13]. A patient with suspected *Candida* pneumonia should have a work-up for right-sided endocarditis.

The only other reported case of isolated pulmonary valve endocarditis due to *Candida* described a 66-year-old man with *C. parapsilosis* fungemia. In that case, a two-stage operation was performed: initial resection of the pulmonary valve leaflets with vegetations and subsequent valve replacement two years later [7]. Current endocarditis guidelines recommend initial or induction therapy with amphotericin B with or without flucytosine with surgical removal of vegetation, followed by chronic suppressive therapy with oral fluconazole [14]. Although he had a large fungal vegetation, our patient did not have any other indications for surgery, such as congestive heart failure, valve dehiscence, or peri-valvular abscess [14]. His chest radiography did not reveal evidence of new septic emboli once therapy was started and his blood cultures sterilized soon after the diagnosis of endocarditis was made. There is conflicting data on outcomes associated with the combined medical and surgical approach versus medical therapy alone [15]. Because patients with *Candida* endocarditis often have other co-morbidities that affect the decision to operate and their outcomes, the optimal management is unclear. However, there have been reports of successful therapy of *Candida* endocarditis with antifungal medical therapy without surgical intervention [16].

Fluconazole and amphotericin B both target ergosterol, a component of the fungal cell membrane. Treating *Candida* endocarditis can be difficult because *Candida* species can form biofilms on native and prosthetic heart valves that can lead to the poor antifungal activity of these agents. The resistance occurs through multiple mechanisms, including decreasing the cell membrane content of ergosterol. The newer antifungal agents, such as amphotericin B lipid formulations and the echinocandins, exhibit novel activity and may be more efficacious against *Candida* biofilms [17]. Combination therapy with echinocandins and lipid formulations of amphotericin B may be able to treat Candida endocarditis while limiting the emergence of resistance [18]. This may obviate the need for surgical removal of the fungal vegetation.

## Conclusion

Persistent *Candida* in respiratory cultures without other etiologies for sepsis may be a sign of right-sided *Candida* endocarditis. Aggressive medical management of this rare condition may be successful with newer antifungal agents that have activity against *Candida* biofilms. Medical therapy without surgical intervention deserves consideration, especially in cases where surgery may not be an option. However, such patients should be monitored closely for signs of treatment failure on medical therapy alone, such as septic embolization.

## Consent

Written informed consent was obtained from the patient for publication of this case report and accompanying images.

## Authors’ original submitted files for images

Below are the links to the authors’ original submitted files for images. Authors’ original file for figure 1 Authors’ original file for figure 2

## Abbreviations

μL: Microliter
mm^3^: Cubic millimeter
PaO_2_: Partial pressure of oxygen
ABG: Arterial blood gas
CT: Computed tomography
HIV: Human immunodeficiency virus
ELISA: Enzyme-linked immunosorbent assay
Mg/dL: Milligram per deciliter
TEE: Trans-esophageal echocardiogram
