# Supplementary figures and images for: Isolated pulmonary valve infective endocarditis in a middle aged man caused by Candida albicans: a case report
Source: BMC Infect Dis. 2014 Oct 30;14:557. doi: 10.1186/s12879-014-0557-5 (PMC4221716; doi:10.1186/s12879-014-0557-5)

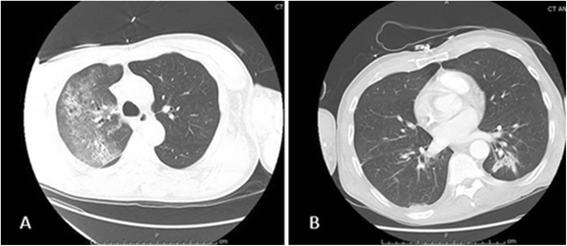

Supplement: Supplementary file 1 — Authors’ original file for figure 1 [file 12879_2014_557_MOESM1_ESM.gif]

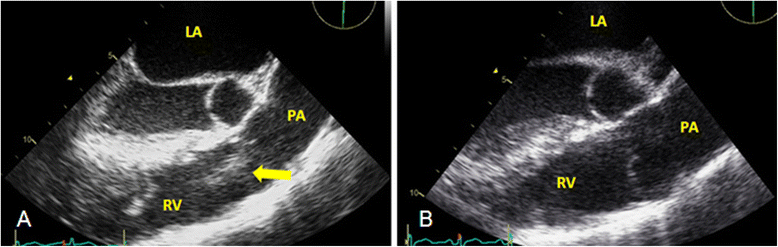

Supplement: Supplementary file 2 — Authors’ original file for figure 2 [file 12879_2014_557_MOESM2_ESM.gif]
